# Appendicular skeletal muscle mass assessment in dogs: a scoping literature review

**DOI:** 10.1186/s12917-022-03367-5

**Published:** 2022-07-16

**Authors:** Ah Young Kim, Lindsay Hochman Elam, Nicolaas Everhardus Lambrechts, Mo D. Salman, Felix Michael Duerr

**Affiliations:** Colorado State University College of Veterinary Medicine and Biomedical Sciences, 300 W Drake Road, Fort Collins, CO 80523 USA

**Keywords:** Muscle mass assessment, Appendicular skeletal mass, Dogs, Scoping review, Skeletal muscle mass

## Abstract

**Background:**

Monitoring changes in appendicular skeletal muscle mass is frequently used as a surrogate marker for limb function. The primary objective of this study was to review scientific information related to the assessment of appendicular skeletal muscle mass in dogs. The secondary objective was to develop practical recommendations for serial evaluation of muscle mass.

**Methods:**

A scoping review was conducted with a systematic search of PubMed, Web of Science, CAB abstract, and Cochrane from inception to June 2021. The following modalities were included in the search: limb circumference, diagnostic ultrasound, computed tomography, magnetic resonance imaging, and dual-energy x-ray absorptiometry.

**Results:**

A total of 62 articles that measured appendicular skeletal muscle mass in dogs were identified. Limb circumference (55 articles) was the most commonly used modality. Its reliability was investigated in five studies. Several factors, including measuring tape type, body position, joint angles, and the presence of hair coat, were reported as variables that can affect measurements. Diagnostic ultrasound (five articles) was validated in three articles, but there is scarce information about observer reliability and variables affecting the measurement. Computed tomography (four articles) and magnetic resonance imaging (one article) have been used to validate other modalities at a single time point rather than as a clinical tool for serial muscle mass monitoring. Dual-energy x-ray absorptiometry (two articles) has been used to quantify specific skeletal muscle mass but was mainly used to evaluate body composition in dogs.

**Conclusion:**

Limb circumference and ultrasound are likely the main modalities that will continue to be used for serial muscle mass measurement in the clinical setting unless a new technology is developed. The reliability of limb circumference is questionable. Several key factors, including measuring tape type, body position, joint angles, and coat clipping, need to be controlled to improve the reliability of limb circumference measurements. Ultrasound may provide a reasonable alternative, but further studies are required to evaluate the reliability of this modality and identify factors that influence ultrasound measurements.

## Background

Skeletal muscle atrophy is a commonly rueported clinical sign in canine veterinary medicine that can be attributed to various conditions, including disuse conditions (e.g., immobilization, inactivity due to pain), neurologic conditions, sarcopenia due to age-related physiologic change in the absence of disease, and cachexia due to systemic conditions (e.g., congestive heart failure, chronic kidney disease, neoplasia) [1, 2]. Monitoring changes in appendicular muscle mass has been frequently used as a surrogate marker for limb function [3], often measured before and after interventions for orthopedic conditions, such as physical therapy [4, 5], total joint replacement [6, 7], tibial plateau leveling osteotomy [8, 9], and fracture repair [10–12] in dogs.

In human medicine, computed tomography (CT) and magnetic resonance imaging (MRI) are considered gold standards for assessing muscle size and cross-sectional area, with dual-energy X-ray absorptiometry (DEXA) considered an alternative [13]. However, the routine use of these modalities in veterinary medicine is problematic for several reasons, including the need for sedation or anesthesia, lack of availability, and relatively high cost. Therefore, an alternative, more widely accessible modality to easily measure limb muscle mass in veterinary patients, is desirable. Limb circumference (LC) may offer such an alternative since it is non-invasive and inexpensive. However, this modality has intrinsic limitations in accuracy for many reasons, including that it measures the muscles indirectly with varying amounts of subcutaneous fat, skin, and hair interposed. Diagnostic ultrasound (US) is a reported alternative that allows non-invasive, safe, and relatively inexpensive visualization of muscle bellies [14].

Even though changes in skeletal muscle mass of limbs have been recognized as an important clinical outcome, a literature review of limb muscle mass measurement with evidence of reliability and validity of modalities in dogs has not been published to date. Therefore, the primary objective of this study was to review scientific information related to the assessment of appendicular skeletal muscle mass in dogs. The secondary objective was to develop practical recommendations for clinical evaluation of muscle mass in the clinical setting. A scoping review was selected to identify the volume of literature and review all relevant evidence [15].

## Materials and methods

This scoping review followed the Preferred Reporting Items for Systematic Reviews and Meta-Analyses Extension for Scoping Reviews (PRISMA-ScR) guidelines [16] and a framework of scoping review suggested by Sargeant and O’Connor [15]:

### Identifying the research question

The following review question, “How have peer-reviewed articles used LC, US, CT, MRI, and DEXA to measure appendicular skeletal muscle mass in dogs?” was formulated using a specific reference population and outcome framework. The population was limited to dogs, and the outcomes included modalities (LC, US, CT, MRI, and DEXA) and their respective methods for appendicular skeletal muscle mass measurement. Those modalities were selected based on accessibility in the veterinary clinical setting from a preliminary search conducted by the primary author (AK).

### Identifying relevant studies

The literature search aimed to identify all relevant citations regarding appendicular skeletal muscle mass measurement using different modalities in dogs. Four online databases, including PubMed, CAB Abstract Complete (1910 to present), Web of Science, and Cochrane, were systematically searched in title and abstract from inception to June 10th 2021.

To identify search terms related to appendicular muscle mass measurement in the database, we searched the Mesh database of PubMed. Combinations of keywords regarding appendicular skeletal muscle mass and modalities were used (Table 1), and Boolean operators AND, OR, and NOT were used to form the combination. Additionally, backward citation tracking as well as a request to experts participating in an internal orthopedic email listserv to identify any missing relevant articles were used. All identified papers from the search were stored in a commercially available reference management software (EndNote, version 20.1).

**Table 1:** Literature search terms

Species	AND	Keywords	AND	Keywords	AND	Modalities
Dog OR Dogs OR Canine		Muscle mass OR Muscle OR Skeletal OR Fat-free mass OR Skeletal muscle OR Lean mass OR Anthropometric OR Body composition OR Limb OR Thigh OR Femoral muscle OR Brachial muscle OR Quadriceps OR Triceps OR Brachium OR Gluteal OR Hamstrings OR Biceps OR Atrophy OR Hypertrophy		Measurement OR Measuring OR Assessment OR Assessing OR Evaluation OR Evaluating		Ultrasound OR Ultrasonography OR MRI OR Magnetic resonance OR CT OR Computed tomography OR Girth OR Circumference OR Dual OR Absorptiometry OR DEXA OR DXA OR Muscle condition score

Searched databases: PubMed, CAB Abstract Complete (1910 to present), Web of Science, and Cochrane

### Study selection

Duplicate citations were removed using the dedicated reference management software function, and the database was then manually reviewed to identify and remove any remaining duplicate citations. All titles and abstracts of the citations were screened by the first author (AK), and those not meeting the following inclusion criteria were excluded:

- The study had to be performed in canines.
- The publication had to be written in English.
- Appendicular skeletal muscle mass had to be measured or estimated by one of the following modalities: LC, US, CT, MRI, or DEXA.
- The study had to measure skeletal muscle mass (e.g., studies measuring the degree of swelling or post-operative edema and studies measuring body composition, such as total lean body mass, fat, and bone mineral density, were excluded).

If it was unclear from the title and abstract whether all criteria were met, full texts were screened. Publications which the title and abstract were in English but the full-text were in a language other than English were excluded. One reviewer (AK) performed the initial screening, and a second reviewer (FD) screened all articles that did not clearly meet the inclusion criteria.

### Data extraction and summation

A data charting form was developed by the first author (AK) using Microsoft Excel® for Mac (version 16.54). The following information was recorded for each study: author, year of publication, modality or modalities used (LC, US, CT, MRI, and DEXA), and the primary purpose of measuring muscle mass in each study (reliability determination, validation, or clinical application). A reliability study was defined as one that evaluated the consistency of the measurement [17], such as assessing intra- and interobserver variability or identifying variables that could affect the measurement. A validation study was defined as a study that compared measurement accuracy to CT or MRI. A clinical application study was defined as a study that measured muscle mass as a clinical outcome measure (e.g., observation of muscle mass change after treatment). Specific details from the materials and methods section were also recorded, including the types of measurement tool, measurement locations, body positions, joint angles, hair coat clipping status, consciousness status (e.g., sedation, anesthesia, or awake), and data collection methods.

## Results

### Study selection

**Fig. 1:**
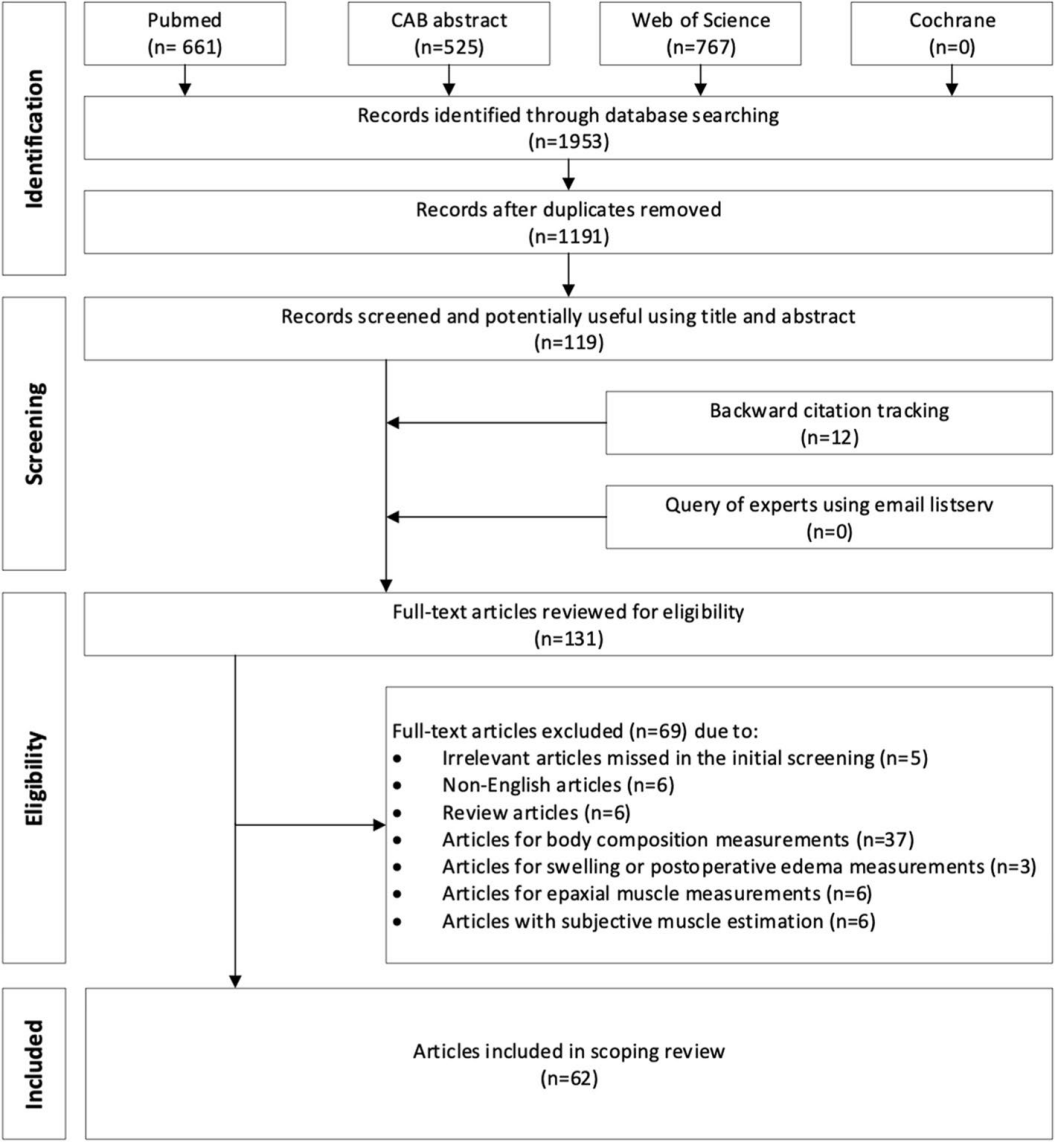
Flow diagram of the selection and screening process

**Fig. 2:**
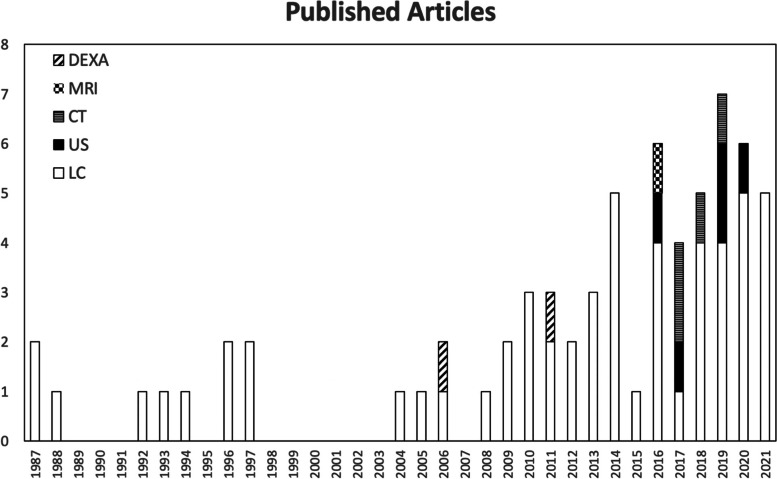
Changes in the volume of literature measuring appendicular skeletal muscle mass over time in each modality. DEXA, Dual-energy x-ray absorptiometry; MRI, Magnetic resonance imaging; CT, Computed tomography; US, Ultrasound; LC, Limb circumference

From the database search, a total of 1953 articles were identified: 661 articles from PubMed, 525 articles from CAB abstract, 767 articles from Web of Science, and 0 articles from Cochrane. After removing duplications, 1191 articles were screened for eligibility. Twelve additional articles were added from the backward citation tracking, and 0 articles were added from the listserv request. The study selection process is demonstrated in Fig. 1. Sixty-two articles were ultimately included in this review spanning from 1987 to 2021. Figure 2 illustrates the growing number of publications over time for each modality.

### Study characteristics

Among the total of 62 qualified articles, LC was used in 55 articles [4–12, 18–63], US in five articles [9, 38, 64–66], CT in four articles [9, 65, 67, 68], MRI in one article [64], and DEXA in two articles [69, 70]. Utilization of the modalities at different time points (i.e., serial measurements) was described in 49 LC articles [4–12, 18–31, 34, 35, 38–43, 45–48, 53–56, 63], two US articles [9, 38], one CT article [9] and one DEXA article [69].

Table 2 outlines the modalities and study classifications. Five studies were classified as reliability studies, and two studies in the validation studies included reliability components (e.g., observer variability). Observer variability was evaluated for LC [44, 50–52] and US [9, 64], which used intraclass correlation coefficient (ICC) [9, 50, 52, 64] and standard deviation [44, 51] for statistical analysis. Table 3 summarizes available observer variability data of LC and US. Measurement variables were evaluated only for LC, including the effect of measuring tape type [44], clipping and sedation [50], sedation or general anesthesia [57], and the effect of stifle angle (e.g., stifle extension, flexion, and standing angle) [50]. Reliability studies for CT, MRI, and DEXA were not available. Three studies were classified as validation studies; correlation between US and MRI in the thigh [64], correlation among LC, US, and CT in the thigh [9], and correlation between US and CT in various locations on the limb [65] have been evaluated. Table 4 summarizes the correlation data. The remaining 54 articles were identified as clinical application studies.

**Table 2:** Classification of the studies based on their purposes of the use of each modality

Purpose of use of modality		LC	US	CT	MRI	DEXA	Number
Reliability study	Baker et al. 2010 [44]	✓^a^					5
	McCarthy et al. 2019 [50]	✓^a^					
	Bascuñán et al. 2016 [51]	✓^a^					
	Smith et al. 2013 [52]	✓^a^					
	Clarke et al. 2020 [57]	✓					
Validation study	Sakaeda et al. 2016 [64]		✓^b, a^		✓		3
	Frank et al. 2019 [9]	✓^b^	✓^b, a^	✓			
	Bullen et al. 2017 [65]		✓^b^	✓			
Clinical application (one modality) [4–8, 10–12, 18–37, 39–43, 45–49, 53–56, 58–63, 66–70]		48	1	2	0	2	54
Clinical application (> two modalities)	White et al. 2020 [38]	✓	✓				
Number of studies		55	5	4	1	2	62

^a^Studies that have evaluated observer variability

^b^Studies that have evaluated the correlation of the modality to CT or MRI

**Table 3:** Observer variability analysis of limb circumference and ultrasound

	Articles	Observers	locations	Intra-observer variability	Inter-observer variability
LC	Baker et al. [44] Standing position 5 dogs 18.1 kg (8.2–24.6 kg)	3	50% thigh	Mean SD = 0.353 ~ 0.569 cm	Mean SD = 1.48 ~ 2.38 cm
			Tibial tuberosity	Mean SD = 0.136 ~ 0.167 cm	Mean SD = 1.02 ~ 1.34 cm
			Hock	Mean SD = 0.369 ~ 0.562 cm	Mean SD = 0.59 ~ 1.35 cm
			Carpus	Mean SD = 0.102 ~ 0.187 cm	Mean SD = 0.46 ~ 0.68 cm
	Bascuñán et al. [51] Standing position Cadavers & 8 Golden Retrievers	4	50% Thigh (cadaver, overall)	Variability±SD 0.09 ± 0.61 cm	Variability±SD 2.26 ± 1.18 cm
			50% Thigh (cadaver, intact hair coat)	–	Variability±SD 2.65 ± 0.65 cm
			50% Thigh (cadaver, shaved hair coat)	–	Variability±SD 2.19 ± 1.19 cm
			50% Thigh (live dog, non-laser guided)	Variability±SD 1.13 ± 0.77 cm	Variability±SD 4.78 ± 2.60 cm
			50% Thigh (live dog, laser guided)	Variability±SD 1.14 ± 0.66 cm	Variability±SD 3.34 ± 1.09 cm
	Smith et al. [52] Lateral recumbency 20 Golden Retrievers 29.1 kg (19.5–37.3 kg)	4	Antebrachium, unknown limb angle	ICC = 0.673 ~ 0.78	ICC = 0.70 ~ 0.72
			Brachium, unknown limb angle	ICC = 0.257 ~ 0.328	ICC = 0.24 ~ 0.38
			Crus, unknown limb angle	ICC = 0.328 ~ 0.703	ICC = 0.42 ~ 0.43
			50% Thigh, unknown limb angle	ICC = 0.222 ~ 0.598	ICC = 0.23 ~ 0.32
	McCarthy et al. [50] Lateral recumbency 10 hound type dogs	2	70% thigh extended	ICC = 0.993, 0.994	ICC = 0.981
			70% thigh standing	ICC = 0.989, 0.991	ICC = 0.972
			70% thigh flexed	ICC = 0.987, 0.992	ICC = 0.973
			50% thigh extended	ICC = 0.986, 0.984	ICC = 0.984
			50% thigh standing	ICC = 0.966, 0.979	ICC = 0.963
			50% thigh flexed	ICC = 0.964, 0.972	ICC = 0.959
US	Frank et al. [9]	1	Pelvic limb muscles	ICC ≥ 0.99	–
	Sakaeda et al. [64]	2	Pelvic limb muscles	ICC = 0.948	–

*ICC* Intraclass correlation coefficient, *SD* Standard deviation

**Table 4:** Correlation of limb circumference and ultrasound data from computed tomography and magnetic resonance imaging data

	Correlations			Locations		Values	Statistical analysis
Bullen et al. [65]	US (MT) vs. CT (MT)			Supraspinatus (*n* = 24)		0.99319	Cronbach’s ɑ ɑ≧0.9: Excellent 0.9 > ɑ≧0.8: Good 0.8 > ɑ≧0.7: Acceptable 0.7 > ɑ≧0.6: Questionable 0.6 > ɑ≧0.5: Poor 0.5 > ɑ: Unacceptable
				Infraspinatus (*n* = 24)		0.98447	
				Cubital extensors (*n* = 15)		0.98901	
				Cubital flexors (*n* = 9)		0.99275	
				Coxofemoral extensors (*n* = 12)		0.73232	
Frank et al. [9]	LC vs. CT (Thigh CSA)			Proximal location		0.77*	Matrix of pseudo R^2^ (* indicates *p*≦0.05)
				Distal location		0.76*	
	LC vs. US (Thigh MT)			Proximal location (lateral side)		0.09*	
				Distal location (lateral side)		0.05	
				Proximal location (medial side)		0.04	
				Distal location (medial side)		0.10*	
	LC vs. US (Rectus femoris CSA)			Proximal location (lateral side)		0.17*	
				Distal location (lateral side)		0.19*	
				Proximal location (medial side)		0.43*	
				Distal location (medial side)		0.27*	
	LC vs. US (Rectus femoris MT)			Proximal location (lateral side)		0.00	
				Distal location (lateral side)		0.00	
				Proximal location (medial side)		0.00	
				Distal location (medial side)		0.00	
	US (Rectus femoris CSA) vs. CT (Thigh CSA)			Proximal location (lateral side)		0.87*	
				Distal location (lateral side)		0.70*	
				Proximal location (medial side)		0.51*	
				Distal location (medial side)		0.80*	
	US (Rectus femoris MT) vs. CT (Thigh CSA)			Proximal location (lateral side)		0.01	
				Distal location (lateral side)		0.19*	
				Proximal location (medial side)		0.04	
				Distal location (medial side)		0.00	
	US (Thigh MT) vs. CT (Thigh CSA)			Proximal location (lateral side)		0.38*	
				Distal location (lateral side)		0.63*	
				Proximal location (medial side)		0.37*	
				Distal location (medial side)		0.03	
Sakaeda et al. [64]	US (MT) vs. MRI (MT) Quadriceps	Proximal 1/6	0.701*	US (MT) vs. MRI (CSA) Quadriceps	Proximal 1/6	0.691*	Intraclass correlation coefficient (* indicates *p*≦0.05, ** indicates *p*≦0.01, *** indicates *p*≦0.001)
		Proximal 1/3	0.857**		Proximal 1/3	0.878***	
		Mid-point	0.751*		Mid-point	0.737*	
		Distal 1/3	0.486		Distal 1/3	–	
		Distal 1/6	–		Distal 1/6	–	
	US (MT) vs. MRI (MT) Biceps femoris	Proximal 1/6	0.385	US (MT) vs. MRI (CSA) Biceps femoris	Proximal 1/6	–	
		Proximal 1/3	0.934***		Proximal 1/3	0.651*	
		Mid-point	0.970***		Mid-point	0.774**	
		Distal 1/3	0.638*		Distal 1/3	0.671*	
		Distal 1/6	–		Distal 1/6	–	
	US (MT) vs. MRI (MT) Semitendinosus	Proximal 1/6	−0.140	US (MT) vs. MRI (CSA) Semitendinosus	Proximal 1/6	–	
		Proximal 1/3	0.790**		Proximal 1/3	0.635*	
		Mid-point	0.653*		Mid-point	0.359	
		Distal 1/3	0.277		Distal 1/3	–	
		Distal 1/6	0.229		Distal 1/6	–	
	US (MT) vs. MRI (MT) Semimembranosus	Proximal 1/6	0.008	US (MT) vs. MRI (CSA) Semimembranosus	Proximal 1/6	–	
		Proximal 1/3	–		Proximal 1/3	–	
		Mid-point	0.543		Mid-point	–	
		Distal 1/3	–		Distal 1/3	–	
		Distal 1/6	0.126		Distal 1/6	–	

*MT* muscle thickness, *CSA* cross-sectional area

### Detailed assessment

#### Limb circumference (55 studies)

Four measuring tape types were described, including standard non-stretchable metric tape, Gulick II tape measure device (Country Technology, Inc., Gays Mills, WI, USA), SECA201 ergonomic measuring tape (Seca North America, Hanover, MD, USA), and QM2000 circumference measuring tape (Quick Medical, Issaquah, WA, USA). When the articles did not specify the type of the measuring tape, it was classified as standard, non-stretchable metric tape. Thirty-two articles used standard non-stretchable metric tape [5, 6, 10–12, 18–43, 63], and 22 articles used Gulick II tape [4, 7–9, 45–62]. One article compared measurements from all four measuring tape types [44].

Several anatomic locations to obtain measurements on the pelvic and thoracic limbs have been described. The most commonly used region was the thigh at a single level [4–12, 18–23, 26–33, 35, 36, 38–42, 44–54, 58–63] but six of these studies [9, 22, 26, 27, 29, 50] also measured the thigh at a second level. Brachium [24, 25, 43, 52, 55, 57], stifle [20, 22, 34, 56, 63], crus [4, 39, 44, 52], and antebrachium [24, 37, 43, 52] circumference at a single level were also described. Specific measurement levels and anatomic landmarks are outlined in Table 5.

**Table 5:** LC measurement locations, landmarks, body positions, and limb angles of limb circumference

**Thigh (47 articles, 53 measurements)**	
Measurement level	• Level of the flank/groin [18, 19], [26]^a^, [27]^a^, [29]^a^, [40] • Proximal 1/4 of the thigh length [21, 23] • Proximal 1/3 of the thigh length [9]^a^, [22]^a^, [35] • Mid-point of the thigh length [5, 6, 8, 11, 12, 20, 31, 36, 38, 44, 47], [50]^a^, [51, 52, 54] • Distal 1/3 of the thigh length [9]^a^, [10], [22]^a^ • 70% of the thigh length from the greater trochanter [4, 45, 46, 49], [50]^a^, [58–62] • 3 cm proximal to the patella [26]^a^, [27]^a^, [29]^a^ • Unspecified location [7, 28, 30, 32, 33, 39, 41, 42, 48, 53, 63]
Proximal landmark	• Greater trochanter [4–6, 8, 9, 20–22, 31, 35, 44–46, 49–52, 54, 58–63] • Unspecified terms, such as flank/groin and ischium
Distal landmark	• Lateral femoral condyle [4, 5, 8, 20, 44–46, 51–54] • Patella [9, 22, 26, 27, 29, 35] • Lateral fabella [49, 50] • Tibial crest [31] • Unspecified terms, such as stifle and thigh
**Brachium (6 articles)**	
Measurement level	• Level of the greater tubercle [55] • Mid-point of the brachium length [52] • Distal 1/3 of the brachium length [24, 25] • 70% of the brachium length from the greater tubercle [57] • Unspecified location [43]
Proximal landmark	• Greater tubercle of the humerus [25, 52, 55, 57] - Superior ridge of the greater tubercle [25] - Cranial/proximal aspect of the greater tubercle [52].
Distal landmark	• Lateral epicondyle of the humerus [25, 52, 57] - Proximal point of the lateral epicondyle [52] - 1 cm below the lateral epicondyle [25] • Unspecified term, humerocubital distance [24]
**Stifle (5 articles)**	
Measurement level	• Immediately below the end of the tibial crest [20] • Proximal part of the patella [22] • Distal part of the patella [56] • Level of plica lateralis [34] • Unspecified location [63]
**Tibia/Crus (4 articles)**	
Measurement level	• Proximal tibia at the level of the greatest width [4] • Proximal aspect of the tibial crest [44] • Distal 1/4 from the lateral femoral condyle to distal point of the lateral malleolus [52] • Unspecified location [39]
**Antebrachium (4 articles)**	
Measurement level	• Proximal 1/4 of the antebrachium length from the lateral humeral epicondyle to proximal point of the styloid process [52] • Proximal 1/3 of the cubitocarpal distance [24] • Mid-point of the carpus and elbow [37] • Unspecified location [43]
**Body positions and limb angles**	
	• Standing body position and standing limb angle [8, 25–27, 32, 33, 35, 36, 44, 46, 51, 53, 55] • Lateral recumbency [4, 5, 9, 19, 22, 38, 50, 52, 54, 57–63] • Standing limb angle [50] • Stifle flexion angle [50] • Stifle extension angle [19, 50, 54, 58–62] • Stifle at 135 ° [9]

^a^Articles that measured the thigh at two levels

The status of the hair coat (clipped or not-clipped) was stated in eight studies [9, 31, 36, 37, 44, 50–52] of the articles. Consciousness status during measurement was stated in nine articles [9, 19, 22, 37, 44, 50, 52, 57, 63] and measurements were performed under sedation [22, 50, 57, 63], under anesthesia [19], and awake [9, 37, 44, 52]. Body position or joint angle during measurement were described in 29 articles and are outlined in Table 5 [4, 5, 8, 9, 19, 22, 25–27, 32, 33, 35, 36, 38, 44, 46, 50–55, 57–63]. Tape tension was not described in any of the papers that used standard, non-stretchable metric tapes; the tension was controlled in papers that used Gulick II tape. Sixteen articles triplicated the measurements to decrease potential intra-observer variability.

Among the 50 clinical application studies, 49 studies serially measured LC as an outcome measure. Both limbs had the same condition (e.g., monitoring muscle mass in patients with hip osteoarthritis) in nine studies, and limbs had various conditions after unilateral procedures (e.g., monitoring muscle mass after fracture repair or TPLO of one side) in the other 40 studies. Those 40 studies used several different methods of data collection, which included presentation of absolute differences (cm, mm) [6, 8, 11, 18, 22, 27, 30, 31, 34, 38, 40, 41, 46, 47, 55] and percentage differences [4, 7, 20, 21, 23, 53] between affected limb and unaffected contralateral limb, absolute differences (cm, mm) [5, 9, 56] and percentage differences [10, 19, 25, 35, 45, 48, 54] between pre-treated and post-treated same single limb, absolute circumference values (cm, mm) of bilateral limbs [39, 43], and normalized limb circumference data by dividing it by the body weight in kilograms [24]. The remaining studies [12, 26, 28, 29, 42, 63] did not clearly state how the comparisons between limbs were made. The nine studies [32, 33, 36, 49, 58–62] that had the same condition between limbs presented absolute circumference values or differences (cm, mm) between limbs of interest.

#### Diagnostic ultrasound (5 studies)

B-mode ultrasound was used in three studies [9, 64, 65], while the remaining two studies did not state the mode. Four studies stated the types of transducer used: 10 MHz linear [38], 12 MHz linear [9], 4–13 MHz linear [65], and 5–8 MHz curvilinear transducer [66]. Four studies described the pressure applied to the transducer, such as ‘the least amount of pressure necessary’ [9], ‘optimal acoustic contact with light manual pressure to minimize muscle compression’ [65], and ‘minimal transducer pressure to minimize tissue distortion’ [66]. Transducer angle was described as either perpendicular to the muscle orientation [64, 65], perpendicular to bone [9, 64, 66], or was not specified [38]. Individual muscles (i.e., supraspinatus [65], infraspinatus [65], quadriceps femoris [64, 66], rectus femoris [9], biceps femoris [64], semitendinosus [64], and semimembranosus [64]) and a group of muscles (i.e., cubital flexors/extensors [65], medial thigh muscles [9], lateral thigh muscles [9, 38], hip flexors [38, 65], and hip extensors [65]) were measured. Multiple levels, described with respect to thigh length, were evaluated only in the thigh, and the measurement locations and landmarks are outlined in Table 6.

**Table 6:** Muscle mass measurement locations and landmarks using ultrasound

**Thoracic limb**	
Measured muscle	• Individual muscle: supraspinatus [65], infraspinatus [65] • Grouped muscles: cubital flexors [65] and cubital extensors [65]
**Pelvic limb: Thigh**	
Measured muscle	• Individual muscle: quadriceps femoris [64, 66], rectus femoris [9], biceps femoris [64], semitendinosus [64], semimembranosus [64] • Grouped muscles: medial thigh muscles [9], lateral thigh muscles [9, 38], cranial thigh muscles (e.g., hip flexors) [38, 65], caudal thigh muscles (e.g., hip extensors) [65]
Measurement level	• Proximal 1/6 of the thigh length [64] • Proximal 1/3 of the thigh length [9, 64] • Mid-point of the thigh length [9, 38, 64, 66] • Distal 1/3 of the thigh length [64] • Distal 1/6 of the thigh length [64]
Proximal landmark	• Greater trochanter [9, 64, 66]
Distal landmark	• Base of the patella [9, 64] • Lateral condyle of the femur [66]

The exact measurement locations of supraspinatus, infraspinatus, caudal thigh muscles, cubital flexors and cubital extensors that Bullen et al. measured are unknown since the paper marked the skin with permanent ink over the regions of interest, not using specific anatomic landmarks [65]

Two studies assessed muscle thickness by measuring the distance between subcutaneous adipose tissue-muscle interface and muscle-bone interface [9, 65], two studies measured the thickness between the superficial and deep outline of the muscle [64, 66], and one study did not specify measurement methods [38]. The cross-sectional area was measured only in one study for the rectus femoris [9].

Hair coat was clipped in 80% [9, 38, 64, 65] of the studies prior to measurements, while the remaining study did not clip the hair coat [66]. Measurements were performed under sedation [9, 64], under anesthesia [65], or awake [38, 66]. Body positions were described as lateral recumbency [38, 64, 66], dorsal recumbency [9], and one article did not specify body position [65]. Joint angles were described as stifle at 135° [9, 64], stifle and tarsus at 90° [38], or not specified [65, 66]. Coxofemoral joint angles were not described in any of the articles.

#### CT (4 studies) and MRI (1 study)

Multiple CT scanners/settings, including 16-slice CT scanner with 0.75 mm slice thickness [67, 68], 64-slice CT scanner with 1 mm slice thickness [65], and unknown CT scanner with 1–2 mm slice thickness [9] were used. Studies used a soft tissue window (width = 350–400 HU (Hounsfield scale), level = 30–40 HU) to evaluate the margins of muscle tissue and a bone window (width = 1500 HU, level = 300 HU) to visualize the bone margin. A 0.3 T MRI with 2 mm sagittal and 4–5 mm transverse slice thickness with T1 weighted or contrast-enhanced T1 weight images was used in one study [64]. Individual muscles (i.e., biceps brachii [67, 68], brachialis [67, 68], supraspinatus [65], and infraspinatus [65]) and a group of muscles (i.e., cubital flexors/extensors [65], hip flexors/extensors [65], and thigh muscles [9]) were measured by CT scan, while individual muscles in the thigh (i.e., biceps femoris, sartorius, semimembranosus, semitendinosus) [64] were measured via MRI. Muscle thickness [64], cross-sectional area of muscle [9, 64, 67, 68], and muscle volume [67, 68] were measured.

Body positions during the CT and MRI scans were described in 80% of the studies, including lateral recumbency [64], dorsal recumbency [9], and ventral recumbency [67, 68]. Joint angles during the scans were only described in two articles [9, 64], namely stifle at 135°. All scans were performed under general anesthesia or deep sedation [9, 64, 65, 67, 68].

#### DEXA (2 studies)

The two available studies utilized a pencil-beam technology [69] or a fan-beam technology [70]. Lean tissue mass of certain sections of the pelvic limbs (i.e., 5 mm slices over the proximal, mid, and distal tibia of both pelvic limbs) [69] and individual muscles (i.e., quadriceps, hamstrings, and gastrocnemius) [70] were measured. Specific details of measurement protocol, including body positions and measurement locations, were described only in one study [69], which was dorsal recumbency with pelvic limbs extended. All scans were performed under general anesthesia.

## Discussion

The present scoping review provides a comprehensive summary describing the clinical use of five modalities (LC, US, CT, MRI, and DEXA) for appendicular skeletal muscle mass measurement in dogs. A scoping review was selected as a review method to provide an overview of the evidence without assessing the risk of bias or methodological limitations, instead of a systematic review that aims to produce a critically analyzed answer to particular questions [71].

The increasing number of publications on the subject over time, as illustrated in Fig. 2, shows the rising application of muscle mass measurement in the clinical and research settings. This review highlights the variability in modalities and measurement protocols selected and the relative popularity of LC compared to other modalities. However, the use of US has increased with all the identified studies published within the past 6 years. As expected, CT and MRI have been used to validate other modalities (i.e., LC and US) for research purposes rather than as a clinical tool for serial muscle mass monitoring. DEXA has been used mainly for evaluating body composition and rarely for quantifying specific skeletal muscle mass in dogs. Unless a new technology is developed or current technological use (e.g., CT and MRI) becomes more accessible, LC and US are likely the main modalities that will continue to be used for serial muscle mass measurement in the clinical setting in the medium term.

When choosing an outcome measure, reliability plays an important role. Understanding variability parameters (e.g., ICC and standard deviation) is essential to interpreting the reliability data of each modality. However, a single variability parameter does not provide enough grounds to judge the reliability of a modality [72]. Unfortunately, all reliability studies included in this review used only one parameter, either ICC or standard deviation of measures. Some studies presented a perspective that ICC solely may not be appropriate for observer reliability calculation due to potential error from a sample size that is small or if values are too homogeneous [72, 73]. A high value of ICC does not always indicate agreement between observers; the number of observers and the difference of actual measurement values need to be considered together. Others suggested that calculating standard deviation is preferred as it visualizes the differences [44, 73]. Therefore, it may be better to present multiple variability parameters for conducting a reliability study of LC or US in the future. Clinicians need to be mindful of interpreting reliability data when utilizing these modalities as clinical or research outcome measures.

The reliability of LC has been a controversial topic since a wide range of ICC has been reported. For example, intra- and interobserver agreement at the mid-thigh level was significantly higher in a study that controlled limb angle (ICC = 0.964–0.986 and 0.959–0.984, respectively) between 2 observers [50] than in a study that did not control limb angle (ICC = 0.222–0.598 and 0.23–0.32, respectively) within four observers [52], both in lateral recumbency. Some readers may have concluded from these studies that LC appears to be a reliable modality when the body position is controlled. However, because the numbers of observers in these two papers are different, these results should be interpreted with caution. From studies that evaluated mean variability±standard deviation, 1.13 ± 0.77 cm of intraobserver variability and 4.78 ± 2.6 cm of interobserver variability were reported in measurements obtained at the mid-thigh level in Golden Retrievers in standing body position [51]. Smaller standard deviations, 0.353–0.569 cm and 1.48–2.38 cm of intra- and interobserver variability, respectively, were noted in smaller dogs at the same level in standing body position [44] as shown in Table 3. The standard deviation of thigh circumference in lateral recumbency has not been published. Combining these results, it is still difficult to conclude the reliability of LC. However, controlling body position and other variables (e.g., hair coat) would be ideal for improving reliability, and the reported standard deviation could be used as a reference for future measurements.

Other essential factors to consider when interpreting observer variability data are observer-blinding methods and body position changes between measurements. Out of four studies that evaluated the observer variability for LC measurements, observers were completely blinded to their measurement values only in two studies by blinding values on the measuring tape [51] or letting assistants read values [50], while observers of the remaining two studies recorded values by themselves at the different time points [44, 52]. Regarding the body position change, only one study let the same dog move around between repeated measurements (e.g., triplicate measurements) of a single observer [52], while the dogs’ body positions (e.g., standing and lateral recumbency) were maintained during the repeated measurements in the remaining three studies [44, 50, 51]. Therefore, to evaluate observer variability that resembles the setting in clinical studies (i.e., a measurement weeks later), future studies may need to consider completely blinding observers to the measurements and letting dogs move around between measurements.

Two studies evaluated the intraobserver variability of US using ICC. Even though the reported intraobserver variability showed good agreement, one cannot judge the reliability of US since only ICC has been reported. Interobserver variability and potential variables (e.g., probe angle and pressure) affecting measurements have not been evaluated to date. User-dependent variations regarding transducer handling have been investigated in human medicine [74, 75]. Muscle thickness was decreased by at least 50% when strong pressure was applied, and a 30° tilt of the transducer elicited up to 15% of the change in the thickness of a flat muscle [74]. However, up to a 6° tilt of the transducer probe was associated with negligible change in the thickness of biceps brachii and tibialis anterior muscles [75]. Additional veterinary research for such variables in US measurements is needed before US can be used more reliably for serial muscle mass measurement in dogs.

The gold standard for measuring appendicular skeletal muscle mass in humans is based on previously reported validity and reliability of CT and MRI [76–78]. Studies evaluating the observer reliability of CT and MRI for appendicular muscle mass measurement in dogs were not identified. Instead, observer variability of those modalities was reported in dogs for assessment of epaxial muscles (e.g., multifidus, semispinalis and longissimus) between two observers with good agreement [79]. Based on the previously reported reliability information from human and veterinary medicine, it is likely that researchers have used CT and MRI for validating other modalities, rather than evaluating their respective reliability.

Combining information from reliability and validation studies may help clinicians to decide which location provides the most consistent measurements. For measuring thigh muscle using LC, McCarthy et al. recommended performing measurements 70% of the distance from the greater trochanter to lateral fabella, with the stifle extended and the dog in lateral recumbency, adding that it was technically easier and more reliable than measuring at 50% of distance because it avoids the flank fold [50]. For measuring thigh muscles using US, Frank et al. suggested that measuring the muscle thickness of the proximal femur (i.e., proximal 1/3 thigh level) on the lateral aspect, which includes quadriceps and biceps femoris muscles, appeared to be the most suitable way for monitoring femoral muscle mass given its close correlation with CT measurements [9]. Sakaeda et al. also showed that individual muscle thicknesses (e.g., biceps femoris, quadriceps, and semitendinosus) at the proximal 1/3 thigh level had good agreement with MRI measurements for these muscles, while measurement of the semimembranosus did not show reliable results. This was thought to potentially be due to its anatomic structure (e.g., no flat interface between muscle and transducer) [64]. Bullen et al. compared hip extensor muscle thickness using US and CT measurements and failed to demonstrate good agreement, but the study did not specify the locations and limb angles [38].

Given the lack of sufficient literature for modalities other than LC, clinical recommendations for serial evaluation were only developed for this modality. Based on the review of the available literature and the authors’ clinical impressions, the following are key considerations that should be considered when selecting LC for appendicular muscle mass measurements:

### Measuring tape

The same type of measuring tape should be used for serial measurements, and ideally, the tension should be controlled. All included studies used the same measuring tape during the study period for serial measurements. The two most commonly used measuring tapes were Gulick II tape and a standard non-stretchable metric tape. Specialized measuring tapes, such as Gulick II, SECA201, and QM2000 (QM2000 tape has been discontinued), have been developed for use in people to provide controlled tension, while the standard non-stretchable metric tape cannot control tension on the object. None of the articles that used the standard non-stretchable metric tape described the tension applied to the tape during the measurements. Baker et al. compared the reliability of the above three specialized measuring tapes and standard non-stretchable metric tape in different locations. Absolute values of the measurements varied by measuring tape type, but all provided consistent measurements.

Interestingly, there was no significant difference in observer variability between the standard non-stretchable metric tape and specialized measuring tapes [44]. Given that the specialized measuring tapes were developed for use in people, it is possible that the degree of tension is not sufficient for subjects with a dense hair coat. It may be necessary to develop a device with greater tension to accommodate for compression of hair coat. Since the study only included a small number of dogs and observers, further research with a large number of dogs and observers is necessary to investigate the most reliable measuring tape type, how much tension should be applied, and how to standardize the tension.

### Measurement locations and landmarks

A specific description of the measurement locations and landmarks should be recorded for serial measurements, and ideally future researchers should utilize the same landmarks. Various bony landmarks have been used, as presented in Table 5. Interestingly, distal landmarks for thigh circumference were variable, including the lateral femoral condyle, base of patella, and lateral fabella. Similarly, some studies specified certain regions of the greater tubercle (e.g., superior ridge, cranial/proximal aspect) and lateral humeral epicondyle (e.g., proximal point, 1 cm below). Likely, researchers have attempted to find more distinct and easily identifiable descriptions for these specific locations, given that the lateral femoral condyle and greater tubercle are ill-defined, relatively large areas. The tibial crest is another ill-defined landmark, given that it is defined as the prominent cranial border of the tibia. There is no study exploring the best landmark for each region, but it would be useful for clinicians to adopt the same landmarks for their location of interest. Even though the lateral femoral condyle and greater tubercle are popular landmarks, we do not believe that those are clearly identifiable. Instead, the lateral fabella and insertion of the infraspinatus muscle on the greater tubercle of the humerus [80] appear to be better landmarks that are not affected by joint motion in similar locations. Based on the evidence in this review and the features of each landmark, we suggest the use of the landmarks outlined in Table 7.

**Table 7:** Recommendation of landmarks to determine the level of limb circumference measurement

Location	Proximal landmark	Distal landmark
**Thigh**	Greater trochanter	Lateral fabella
**Crus**	Tibial tuberosity	Lateral malleolus of the fibula
**Brachium**	Insertion of the infraspinatus muscle on the greater tubercle of the humerus	Lateral epicondyle of the humerus
**Antebrachium**	Lateral epicondyle of the humerus	Styloid process of the ulna

There have been several efforts to mark the location for consistent measurement by using a marker [50], laser guidance [52], or permanent tattoos at a landmark [45]. Bascuñán et al. reported that laser guidance at the mid-thigh in the standing position improved inter-observer variability but did not impact intra-observer variability [52]. Therefore, if multiple observers perform measurements, this technology may be considered. Marking the measurement location may be unacceptable to dog owners participating in clinical trials, but could be considered in research studies.

### Status of hair coat

Hair coat status needs to be identical between measurements, and ideally, the hair coat should be clipped short at the measuring site. Based on the available literature, hair coat clipping appears to be a significant factor influencing observer variability. Bascuñán et al. showed a significant difference (3.44 ± 1.31 cm difference, *p* < 0.001) of thigh circumference between clipped and unclipped limbs among five long-haired, large breed canine cadavers [52]. McCarthy et al. did not show a statistical difference in thigh circumference measurement before and after clipping, but average differences were 3 mm (pre-clipping: 33.9 ± 2.6 cm, post-clipping: 33.6 ± 1.8 cm) and 7 mm (pre-clipping: 38.8 ± 2.7 cm, post-clipping: 38.1 ± 3.1 cm) at the 70 and 50% thigh location, respectively, in 10 hound-type mixed breed dogs [50]. The different dog breeds (i.e., long-haired large breed and hound-type mixed breed) of those two papers might explain the discrepancy in the results. White et al. mentioned hair regrowth after TPLO as a potential reason for their thigh circumference results differing between LC and US thickness measurements [38]. Unfortunately, only eight articles included in this review stated the status of hair coat clipping. Until definitive research is available, hair coat length should ideally be controlled when performing serial measurements.

### Body position and limb angles

Body position and limb angles of all joints of the limb need to be maintained at consistent angles when performing serial measurements. It was surprising that only 52% of published studies stated limb angles or body positions because these variables significantly impact LC [50]. Reported body positions were either standing or laterally recumbent, and there is no available research that determines which body position provides more consistent measurements. In lateral recumbency, the impact of stifle angles (e.g., extension, flexion, standing) was investigated, and stifle extension provided more consistent measurements for the thigh [50]. The influence of other joint angles, such as the coxofemoral joint, has not been reported. However, it is reasonable to assume that, unless proven otherwise, all joint angles should be controlled. This is also relevant when utilizing advanced imaging (e.g., CT or MRI), which are considered gold standards in people. When utilizing these modalities for serial measurements, attention must be paid to maintaining the same position during scans [9].

### Consciousness status

Serial measurements should be performed under the same state of consciousness, particularly in anxious dogs. About 16% of articles described details of the consciousness status. Based on the available literature, sedation and anesthesia may not affect serial measurements in calm dogs. McCarthy et al. showed a statistically insignificant decrease in thigh circumference after sedation in calm dogs placed in lateral recumbency [50]. Similarly, Clarke et al. also showed a slight decrease in thoracic limb circumference after sedation/general anesthesia compared to fully conscious, calm dogs in lateral recumbency, without statistical significance [57]. Both studies suspected that the slight decrease in the value might be due to muscle relaxation. Even though sedation/anesthesia status may not significantly impact the measurements in calm dogs, we still recommend measuring LC under a consistent state of consciousness, particularly in anxious/active dogs. Since most clinical recheck examinations may not require sedation, measuring the circumference before treatments without sedation or anesthesia may be ideal (i.e., when performing a study that utilizes LC after stifle surgery, consider performing the pre-operative measurements prior to sedation).

### Collecting and comparing measurements

The reported data should include absolute values and a detailed description of the study population. The presentation of limb circumference data has been inconsistent in the veterinary literature. This is particularly evident in studies investigating muscle mass change after unilateral procedures (e.g., monitoring muscle mass after fracture repair or TPLO). Some researchers presented the absolute (i.e., change in mm) or relative differences (i.e., percentage change) between the affected and unaffected contralateral limb, while others utilized the treated limb over time. Regardless of which limb is chosen as the control, including absolute values allows for a more transparent estimation of actual change than limiting the reported data to relative values. A detailed description of the included dog characteristics (i.e., weight, conformation, BCS, and breed) is required. Then, if future studies were limited to certain breeds, the previously published absolute values could be combined in future analyses.

The present review has several limitations. First, its search strategy may not have included all muscle mass measurement tools and articles written in languages other than English. In human medicine, other measurement modalities, such as quantitative magnetic resonance [81] and bioelectrical impedance analysis [82], are being used to estimate appendicular skeletal mass. Second, all studies including low-quality evidence (e.g., case reports and case series) were included since scoping reviews collect information from a broad range of studies and rarely assess the quality of evidence. Third, some articles may have been missed if they did not include their modalities in their titles or abstracts. While we implemented strategies to address this concern, such as backward citation tracking, some manuscripts may not have been identified.

## Conclusions

The assessment of skeletal muscle mass provides an important functional evaluation of the canine patients. CT and MRI can measure muscle mass accurately at a single time point, which is ideal for comparing measurements at the same location (i.e., comparing left and right thigh muscles). However, those modalities are difficult to use routinely in dogs to measure muscle mass change over time due to the cost, operational complexities, and requirement of sedation or anesthesia. LC and US are non-invasive and inexpensive modalities that can be easily used serially to monitor muscle mass change in the clinical setting. LC has been most frequently utilized, but its reliability is questionable. Based on the analysis of the reviewed articles, several factors, including measuring tape type, body position, joint angles, and coat clipping, need to be controlled to improve the reliability of the measurement. The use of US appears to be gaining popularity, but there are few reliability studies that examined observer variability and variables affecting measurements. Further research is required to provide clinical recommendations for US. This scoping review provides key considerations for using LC and reveals several future research topics for measuring appendicular skeletal muscle mass in dogs.

## Data Availability

The datasets used and/or analyzed during the current study are available from the corresponding author on reasonable request.

## Abbreviations

LC: Limb circumference
US: Diagnostic ultrasound
CT: Computed tomography
MRI: Magnetic resonance imaging
DEXA: Dual-energy X-ray absorptiometry
ICC: Intraclass correlation coefficient
SD: Standard deviation
MT: Muscle thickness
CSA: Cross-sectional area
